# Fc gamma receptor IIb participates in maternal IgG trafficking of human placental endothelial cells

**DOI:** 10.3892/ijmm.2015.2141

**Published:** 2015-03-17

**Authors:** TOMOKO ISHIKAWA, TAKAMI TAKIZAWA, JUN IWAKI, TAKUYA MISHIMA, KUMIKO UI-TEI, TOSHIYUKI TAKESHITA, SHIGEKI MATSUBARA, TOSHIHIRO TAKIZAWA

**Affiliations:** 1 Department of Molecular Medicine and Anatomy, Nippon Medical School, Tokyo 113-8602, Japan; 2 Department of Biological Sciences, Graduate School of Science, University of Tokyo, Tokyo 113-0033, Japan; 3 Department of Obstetrics and Gynecology, Nippon Medical School, Tokyo 113-8602, Japan; 4 Department of Obstetrics and Gynecology, Jichi Medical University, Tochigi 329-0498, Japan

**Keywords:** Fcγ region receptor IIb, maternal IgG, human placenta, placental endothelial cells, RAS-related protein Rab-3D

## Abstract

The human placental transfer of maternal IgG is crucial for fetal and newborn immunity. Low-affinity immunoglobulin gamma Fc region receptor IIb2 (FCGR2B2 or FcγRIIb2) is exclusively expressed in an IgG-containing, vesicle-like organelle (the FCGR2B2 compartment) in human placental endothelial cells; thus, we hypothesized that the FCGR2B2 compartment functions as an IgG transporter. In this study, to examine this hypothesis, we performed *in vitro* bio-imaging analysis of IgG trafficking by FCGR2B2 compartments using human umbilical vein endothelial cells transfected with a plasmid vector containing enhanced GFP-tagged FCGR2B2 (pFCGR2B2-EGFP). FCGR2B2-EGFP signals were detected as intracellular vesicular structures similar to FCGR2B2 compartments *in vivo*. The internalization and transcytosis of IgG was significantly higher in the pFCGR2B2-EGFP-transfected cells than in the mock-transfected cells, and the majority of the internalized IgG was co-localized with the FCGR2B2-EGFP signals. Furthermore, we isolated FCGR2B2 compartments from the human placenta and found that the Rab family of proteins [RAS-related protein Rab family (RABs)] were associated with FCGR2B2 compartments. Among the RABs, RAB3D was expressed predominantly in placental endothelial cells. The downregulation of *RAB3D* by small interfering RNA (siRNA) resulted in a marked reduction in the FCGR2B2-EGFP signals at the cell periphery. Taken together, these findings suggest that FCGR2B2 compartments participate in the transcytosis of maternal IgG across the human placental endothelium and that RAB3D plays a role in regulating the intracellular dynamics of FCGR2B2 compartments.

## Introduction

Immunoglobulin G transport from the maternal circulation to the fetus in the human placenta is crucial for the immunity of the fetus and the newborn, whose own immune system is maturing. This mechanism, which is not yet well understood, requires IgG movement across two cell layers of the human placenta: the syncytiotrophoblast and the fetal placental endothelium (1,2). Transport across the syncytiotrophoblast is thought to be mediated by endosomes containing the MHC-related Fc receptor, FCGRT (also known as FcRn) (3,4). An efficient transcytotic mechanism to move the IgG from the apical to the basolateral surface of the syncytiotrophoblast via the FCGRT-containing endosomes can readily be envisioned, but has not yet been substantiated. The mechanisms through which IgG crosses the fetal endothelium are much less clear.

Low-affinity gamma immunoglobulin Fc region receptor IIb (FCGR2B; also designated as FcγRIIb) is a negative regulator of immune cell function that is encoded by a single *FCGR2B* gene; the two most common isoforms, B1 (FCGR2B1) and B2 (FCGR2B2), are generated by the alternative splicing of the corresponding mRNA sequences (5). FCGR2B is generally expressed on the surface of immune cells, e.g., neutrophils, B-lymphocytes and monocytes (6,7), but is not expressed in any other endothelial cells of the adult human body, apart from placental endothelial cells (8) and hepatic sinusoidal endothelial cells (9). Human placental endothelial cells abundantly and predominantly express FCGR2B2 (10–12). We previously identified an FCGR2B2-defined, IgG-containing organelle (tentatively designated as the FCGR2B2 compartment) in placental endothelial cells by immunoelectron microscopy; the FCGR2B2 compartments did not overlap with various marker proteins of well-recognized intracellular organelles [e.g., caveolae, secretory granules and (early, late and recycling) endosomes] (11). These previous *in vivo* findings suggested that FCGR2B2 compartments mediate the transfer of IgG across the placental endothelium, independent of caveolae. However, the molecular mechanisms underlying the formation and intra cellular dynamics of FCGR2B2 compartments and their IgG trafficking in placental endothelial cells remain to be elucidated.

In this study, we performed bio-imaging analysis of IgG trafficking of FCGR2B2 compartments using human umbilical vein endothelial cells (HUVECs) transfected with a plasmid vector containing enhanced GFP-tagged FCGR2B2 (pFCGR2B2-EGFP) as an *in vitro* system for the analysis of FCGR2B2 expression in placental endothelial cells. We also isolated FCGR2B2 compartments from the human placenta and performed proteomic analysis of the vesicles to identify the molecules involved in the regulation of the FCGR2B2 compartment trafficking; we found that the Rab family of proteins [RAS-related protein Rab family (RABs)] were associated with FCGR2B2 compartments in placental endothelial cells. Among the RABs, RAB3D was expressed predominantly in placental endothelial cells. Furthermore, we generated small interfering RNAs (siRNAs) targeting *RAB3D* to investigate the role of the RAB3D in the FCGR2B2 compartment in our *in vitro* system.

## Materials and methods

### Sample collection

Human first-trimester placentas and full-term placentas with umbilical cords from patients who provided informed consent were obtained according to the protocols approved by the Nippon Medical School Hospital Ethics Committee (Tokyo, Japan) and the Jichi Medical University Ethics Committee (Tochigi, Japan). Tissue samples were processed as soon as possible following delivery (within 20 min).

### Isolation of endothelial cells from the human placenta

Human umbilical cords were processed to obtain the HUVECs through collagenase digestion and subsequent magnetic bead isolation (Dynabeads CD31; catalog no. DB11128; Invitrogen, Carlsbad, CA, USA). The HUVECs were maintained with the endothelial cell growth medium MV2 kit (catalog no. C-22121; PromoCell, Heidelberg, Germany) at 37°C in a humidified incubator with 5% CO_2_. The placental endothelial cells were isolated from human placental tissues as described in a previous study (3). Dynabeads CD31 was used instead of Dynabeads that were coated with QB-End/40 monoclonal antibody to thrombomodulin.

### Plasmid construction and transient transfection by electroporation

The open reading frame of human *FCGR2B2* cDNA was amplified as previously described (12). The PCR product was inserted at the *Hin*dIII and *Age*I sites of a pEGFP-Hyg-N1 vector, as previously described (13), which was generated by modifying the commercially available pDsRed-Monomer-Hyg-N1 vector (BD Biosciences Clontech, Mountain View, CA, USA). These plasmids were designed to express EGFP fused to the C-terminus of the FCGR2B2 protein avoiding the signal peptide. The final construct was designated as pFCGR2B2-EGFP. A pFCGR2B2 vector lacking EGFP was also constructed by the PCR method and used to provide experimental foundations for the bio-imaging analysis of IgG trafficking using HUVECs transfected with pFCGR2B2-EGFP. As a mock control, cells were transfected in parallel with vector pEGFP-N1.

For transfection, 100 *μ*l HUVECs were trypsinized, resus-pended with serum-free Opti-MEM (Invitrogen) at a density of 1×10^6^ cells/ml, and placed in 2 mm cuvettes with 2 *μ*g plasmid. Electroporation was performed using an exponential wave pulse of 100 V and 500 *μ*F using the Gene Pulser Xcell electroporation system (Bio-Rad, Hercules, CA, USA). The the cells were then replated with 200 *μ*l medium and cultured for up to 72 h. Validation of our plasmid construction and transfection procedure was performed by transfection of the HUVECs with pFCGR2B2-EGFP followed by immunocytochemistry.

### Antibodies

**Figure 1:**
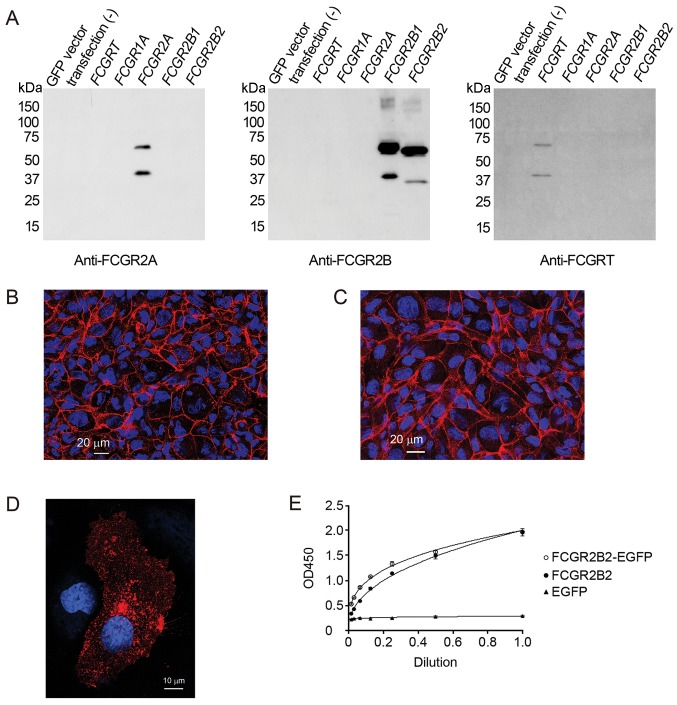
Validation of antibodies and characterization of pFCGR2B2-EGFP-transfected human umbilical vein endothelial cells (HUVECs). (A) Validation of antibodies. The anti-FCGR2A, anti-FCGR2B and anti-FCGRT antibodies used in this study were validated by western blot analysis. Lysates of Cos-7 cells transfected with a plasmid designed to overexpress EGFP or EGFP-tagged FCGRT, FCGR1A, FCGR2A, FCGR2B1 or FCGR2B2 were used for this validation. The upper band in each lane corresponds to an EGFP-conjugated Fc receptor and the lower band corresponds to an Fc receptor without EGFP. (B and C) Representative immunofluorescence images of CD31 and CD144 in HUVECs plated on cell culture inserts. (B) CD31 and (C) CD144 are localized along the lateral membrane of adjacent cells. (D and E) Comparison of the subcellular localization and IgG binding property of FCGR2B2-EGFP with those of FCGR2B2. (D) Representative immunofluorescence image of a pFCGR2B2-transfected HUVEC. FCGR2B2 signals are detected as intracellular vesicular and tubular structures; some signals are also present on the cell surface. Note that a pFCGR2B2-EGFP-transfected cell (Fig. 2A) shows similar localization of FCGR2B2 in the pFCGR2B2-transfected cell. (E) The affinities of human IgG for FCGR2B2-EGFP and FCGR2B2 detected by enzyme-linked immunosorbent assay (ELISA). FCGR2B2-EGFP (white circles) shows an almost identical affinity for FCGR2B2 (black circles). By contrast, EGFP from mock-transfected pEGFP-N1 cells do not exhibit selectivity for IgG (black triangles). Three independent experiments were performed; error bars represent the means ± SD. FCGR2B2, low-affinity immunoglobulin gamma Fc region receptor IIb2.

Rabbit anti-human FCGR2B antibody directed against the C-terminal cytoplasmic tail of human FCGR2B (MHPDALEEPDDQNRI) and rabbit anti-human FCGRT antibody (DADLKDVNVIPATA) directed against the C-terminal cytoplasmic tail of human FCGRT were generated (Peptide Institute, Osaka, Japan). Rabbit anti-human low-affinity immunoglobulin FCGR2A antibody directed against the C-terminal cytoplasmic tail of human FCGR2A (YLTLPPNDHVNSNN) was also generated and used to test the specificity of the anti-FCGR2B antibody. Cos-7 cells (Cell Resource Center for Biomedical Research Institute of Development, Aging and Cancer Tohoku University, Miyagi, Japan) were transfected with a plasmid designed to overexpress EGFP or EGFP-tagged FCGRT, FCGR1A, FCGR2A, FCGR2B1 or FCGR2B2; the specificity of these affinity-purified antibodies was validated by western blot analysis (Fig. 1A). Rabbit anti-von Willebrand factor antibody (catalog no. F3520; Sigma-Aldrich), mouse anti-caveolin-1 antibody (catalog no. C-37120-150; BD Transduction Laboratories, Franklin Lakes, NJ, USA), mouse anti-CD31 antibody (catalog no. BBA7; R&D Systems, Minneapolis, MN, USA), mouse anti-CD144 (clone 16B1; eBioscience, San Diego, CA, USA; CD144 also known as VE-cadherin and CDH5), rabbit anti-human RAB1 antibody (catalog no. FL-205), rabbit anti-human RAB3 antibody (catalog no. FL-220) (both from Santa Cruz Biotechnology, Inc., Santa Cruz, CA, USA), rabbit anti-human RAB3D (catalog no. 12320-1-AP; ProteinTech Group, Chicago, IL, USA), mouse anti-human RAB11 antibody (catalog no. 610656; BD Transduction Laboratories), mouse anti-human RAB33B (catalog ID H00083452-M01; Abnova, Taipei, Taiwan), mouse anti-human Ras-related protein Ral-A antibody (catalog no. 610221; BD Transduction Laboratories), Alexa Fluor-labeled secondary antibodies (Invitrogen), and HRP-conjugated secondary antibodies (Jackson ImmunoResearch Laboratories, West Grove, PA, USA) were obtained from commercial sources.

### Immunocytochemistry

The cultured HUVECs were fixed with 4% paraformaldehyde in PBS at room temperature for 2 h and permeabilization was performed with 0.2% Triton X-100. For the detection of caveolin-1, additional permeabilization was performed with 0.5% sodium dodecyl sulfate (SDS). Dilutions for the primary antibodies were 1:500 (FCGR2B), 1:200 (von Willebrand factor), 1:100 (caveolin-1), 1:500 (CD31), and 1:20 (CD144). Alexa Fluor-labeled secondary antibodies were used at 1:200 dilutions. Following incubation with the antibodies, the cells were subjected to nuclear staining with 4',6-diamidino-2-phenylindole (DAPI) for 10 min and were mounted in ProLong Gold (Invitrogen). Fluorescence images were collected using a BX60 microscope (Olympus, Tokyo, Japan) equipped with a Spot RT SE6 CCD camera (Diagnostic Instruments, Sterling Heights, MI, USA) and captured with the MetaMorph image analysis system (Universal Imaging, Dowingtown, PA, USA). The figures were compiled using Photoshop CS software (Adobe Systems, Mountain View, CA, USA).

### Binding affinity of FCGR2B2-EGFP for human IgG

The binding property of FCGR2B2-EGFP for human IgG was analyzed by enzyme-linked immunosorbent assay (ELISA), as previously described (14,15). Rabbit anti-human FCGR2B antibody was biotinylated using a Biotin Labeling kit-NH_2_ (Dojindo Molecular Technologies, Kumamoto, Japan). MaxiSorp 96 well microtiter plates (Nunc, Roskilde, Denmark) were coated with 100 *μ*g/ml human IgG (catalog no. 31154; Thermo Fisher Scientific Pierce, Waltham, MA, USA) at 4°C overnight. The plates were blocked with 5% non-fat dry milk in PBS containing 0.05% Tween-20 (PBST) for 1 h at room temperature and then incubated with serially diluted cell lysate samples from the transfected HUVECs for 2 h at 37°C. After washing with Tween-20-containing PBS, the plates were incubated with the biotinylated anti-FCGR2B antibody at 1:1,000 dilutions for 2 h at 37°C. High Sensitivity Streptavidin HRP conjugates at 1:10,000 dilutions (catalog no. 21130; Thermo Fisher Scientific Pierce) was added to each well followed by incubation for 1 h at 37°C. ELISA was developed using 1-Step Ultra TMB-ELISA (Thermo Fisher Scientific Pierce,). The absorbance of each well was measured at 450 nm using an iMark Microplate Absorbance Reader (Bio-Rad).

### Incorporation of human IgG and fluorescence imaging

We evaluated the ability of the pFCGR2B2-EGFP-transfected HUVECs to incorporate IgG using a modification of a previously described method (16,17). Alexa 555-human IgG and Alexa 633-human IgG were prepared using human IgG (Thermo Fisher Scientific Pierce) and an Alexa Fluor protein labeling kit (Invitrogen). Alexa 555-F(ab′)_2_ was generated by digesting Alexa-human IgG using a Pierce F(ab′)_2_ Preparation kit (catalog no. 44988; Thermo Fisher Scientific Pierce) and purified by chromatography and dialysis using the methods recommended by the manufacturer.

Preliminary experiments of the cells exposed to different concentrations of Alexa 555-IgG or Alexa 555-F(ab′)_2_ (0, 1, 10 and 100 *μ*g/ml) indicate that the minimum concentration of detectable fluorescence signals in imaging analysis was 10 *μ*g/ml. After 24 h transfection with pFCGR2B2-EGFP or pEGFP-N1, HUVECs were washed with HBSS (catalog no. H4891; Sigma-Aldrich) containing 10 mM HEPES (pH 7.4), serum starved for 20 min, and then exposed to 10 *μ*g/ml Alexa 555-IgG or equimolar Alexa 555-F(ab′)_2_ for 10 min. The cells were immediately washed with PBS and incubated with fresh medium for another 10 min. For quantitative image analysis of Alexa 555-IgG and Alexa 555-F(ab′)_2_, the cells were fixed with 4% paraformaldehyde, counterstained with DAPI, and mounted in ProLong Gold. The signals were detected using a fluorescence microscope (IX71; Olympus) or a confocal laser-scanning microscope (LSM 710; Carl Zeiss, Jena, Germany).

The quantitative image analysis of the pFCGR2B2-EGFP-transfected or mock-transfected HUVECs was performed using the MetaMorph image analysis system as follows: using the auto-fluorescence threshold on merged images, the cellular profiles were demarcated as a region of interest. The area within the region of interest occupied by the fluorescence signals of the internalized Alexa 555-IgG or Alexa 555-F(ab')_2_ were detected with an appropriate fluorescence threshold. Approximately 120 cells were used for this analysis. The figures were compiled using Photoshop CS software.

The ability of IgG to incorporate from the basolateral surface was evaluated as follows: the HUVECs transfected with pFCGR2B2-EGFP were plated on Transwell membrane inserts for 24-well plates (0.4 *μ*m pores, catalog no. 353495, BD Falcon, Franklin Lakes, NJ, USA) at a density of 2×10^5^ cells/insert and cultured for 24 h. Confluent HUVECs were washed with HBSS containing 10 mM HEPES (pH 7.4) and serum starved for 20 min. The Alexa 633-IgG (100 *μ*g/ml) was added to the wells of the lower chambers of the 24-well plates. Following incubation for 60 min, the cells were immediately washed and fixed. Super-resolution microscopy detection of FCGR2B2-EGFP and Alexa 633-IgG incorporation was performed using a stimulated emission-depletion (STED) microscope (TSC STED CW; Leica, Wetzlar, Germany). Images were compiled using Photoshop CS software.

### Transcytosis of human IgG

We evaluated the ability of the pFCGR2B2-EGFP-transfected HUVECs to transcytose IgG using a modification of a previously described method (18,19). Human IgG (Thermo Fisher Scientific Pierce) was biotinylated using a Biotin Labeling kit-NH_2_. The HUVECs transfected with pFCGR2B2-EGFP or pEGFP-N1 were plated on Transwell membrane inserts for 24-well plates at a density of 1×10^5^ cells/insert and cultured for 24–36 h.

To confirm the confluence of the polarized monolayers, we validated the passage of Lucifer yellow and the formation of cell-cell junctions (Fig. 1B and C). Before conducting IgG transcytosis experiments, 100 *μ*M Lucifer yellow (catalog no. L-1177; Invitrogen-Molecular Probes, Eugene, OR, USA) was added to the basolateral chamber of HUVECs growing on the culture inserts. After 4 h, the concentration of Lucifer yellow of apical media in the culture inserts was measured using a GloMax 96 microplate luminometer (Promega, Madison, WI, USA). The integrity of the monolayer was confirmed with <2% leakage of Lucifer yellow flux observed in all the experiments. Immunofluorescence analysis revealed that adherens junction (CD144) and adhesive molecule (CD31) were expressed on cell to-cell contacts. These results indicated that the HUVECs developed a confluent polarized monolayer.

After testing the cell monolayer integrity, 15 *μ*g/ml biotin-labeled human IgG were added to in the basolateral donor chamber. After 24 h, transcytosed, biotin-labeled human IgG in the apical receiver chamber was detected by western blot analysis using high sensitivity streptavidin HRP conjugates at 1:10,000 dilutions (catalog no. 21130; Thermo Fisher Scientific Pierce). Signal detection was carried out using Immobilon Western HRP Substrate (Millipore, Billerica, MA, USA) and an LAS-4000 lumino-image analyzer (Fujifilm, Tokyo, Japan). Densitometric analysis of the band intensities of the heavy chain of the transcytosed, biotin-labeled IgG was quantitatively analyzed using Multi Gauge software (GE Healthcare, Little Chalfont, UK).

### Gene silencing

The siRNA-mediated knockdown of *FCGRT* and *RAB3D* in the HUVECs was performed as follows: two distinct types of siRNA duplexes for each target gene were designed using siDirect (http://sidirect2.rnai.jp/) as previously described (20), which is based on an algorithm to increase the knockdown efficiency and minimize off-target silencing. The designed siRNAs were synthesized by Nippon EGT (Toyama, Japan). The transfection of the siRNAs was performed using Lipofectamine 2000 (Invitrogen) according to the manufacturer’s instructions. The efficacy of gene silencing at the mRNA level was assessed after 6–72 h by reverse transcription-quantitative polymerase chain reaction (RT-qPCR). The sequences of the designed siRNAs were as follows: siRNAs for *FCGRT*, si*FCGRT*-1 [21 nt guide (5′→3′): acuuuugacuguua gugacGA; 21 nt passenger (5′→3′): gucacuaacagucaaaaguGG)] and si*FCGRT*-2 [21 nt guide: uuuacauccuucaaa ucagCA; 21 nt passenger: cugauuugaaggauguaaaUG)]; siRNAs for *RAB3D*, si*RAB3D-1* [21 nt guide: uaucaguagcaguuugaacAU; 21 nt passenger: guucaaacugcuacugauaGG)] and si*RAB3D-2* [21 nt guide: acauugugaaggaaugagcCA; 21 nt passenger: gcucauuccuu cacaauguCG)]; control siRNA [21 nt guide: uucuccgaacguguc acguTT; 21 nt passenger: acgugacacguucggagaaTT)]. Nucleotides shown by uppercases indicate those of 3′-overhangs.

### Western blot analysis

Proteins in the HUVECs were extracted using M-PER Mammalian Protein Extraction Reagent (Thermo Fisher Scientific Pierce) containing protease inhibitors. Proteins of isolated FCGR2B2 compartments were directly extracted with SDS-containing loading buffer. Dilutions for the primary antibodies were 1:5,000 (FCGR2B, Ral-A and ACTB), 1:250 (FCGRT), 1:200 (RAB1 and RAB3), 1:400 (RAB3D) and 1:1,000 (RAB11 and RAB33B). HRP-conjugated secondary antibodies were diluted to 1:10,000. Signals were detected using Immobilon reagent and visualized using a LAS-4000 Lumino image analyzer. The intensity of the visualized signals was quantitatively analyzed using Multi Gauge software.

### RT-qPCR

Total RNA was extracted from the samples using Isogen reagent (Nippon Gene, Tokyo, Japan) according to the manufacturer's instructions. cDNA synthesis was performed using a PrimeScript RT reagent kit (Takara Bio, Shiga, Japan). For conventional RT-PCR, ExTaq Hot Start Version (Takara Bio) was used for amplification using gene-specific primers, some of which were designed using DNASIS software (Hitachi, Tokyo, Japan).

The primers used were as follows: *FCGR2B* forward, CCACTAATCCTGATGAGGCTGACA and reverse, CTC AAATCCCAATGCAAGACAATG; *FCGRT* forward, CAC GCCTCGTCGTCACTAACA and reverse, AGTAGCAAG ACACCGATGACGATTC; *RAB1A* forward, TATGGGACA CAGCAGGCCAGG and *r*everse, ACGGAATTCCAAGGG AATCAGC; *RAB1B* forward, TGAACCCCGAATATGAC TAC and reverse, GTGTACGTGTCATCAGCAAA; *RAB3A* forward, CAGACCTGTTCTGACCTCAT and reverse, CTTTATTGGGTGCGTGTAGT; *RAB3B* forward, AGATGGTCCCAGTAATAGATACTC and reverse, GACTGTTCTCT AAGTCCCTGTAGT; *RAB3C* forward, GCGACAAAATGT CAGAGAGT and reverse, GAGGAGGAGTTTCCTTGAGT; *RAB3D* forward, TCTGGAACTATGGACCACAT and reverse, CTCCTGGCTCTGAGGTTAAT; *RAB2A* forward, CAGGTGTTGGTAAATCATGC and reverse, CACCGAA CTCTACACCAATAG; *RAB15* forward, ATCAAGACC TATGCCACATC and reverse, CCACTGCCCAATATAA CTTC; *RAB18* forward, TACCCC TCAGTAAGATTCCA and reverse, TATTGCAAAGGTGGTCACTC; *RAB35* forward, CCAGGATGTGTTTCCTTAGA and reverse, AACTGCA GTGTGATCTGTGA; *RAB39A* forward, ACGTCAAGTT ACAAGGGAAG and reverse, TGTCTCTCGTCAAGATTGTG; *ACTB* forward, ATTGCCGACAGGATGCAGA and reverse, GAGTACTTGCGCTCAGGAGGA; and *GAPDH* forward, GCACCGTCAAGGCTGAGAAC and reverse, ATG GTGGTGAAGACGCCAGT. All primers were synthesized by Nippon EGT. The PCR products were separated by electrophoresis on a 2% agarose gel. Quantitative PCR (qPCR) was performed using SYBR Premix ExTaq (Takara Bio) on an ABI 7300 apparatus (Applied Biosystems, Foster City, CA, USA). *GAPDH* was used for the ormalization of gene expression.

### Isolation of FCGR2B2 compartments from the human placenta

FCGR2B2-containing vesicles were purified from full-term placentas as follows: human placental terminal villus-rich fractions were collected from full-term placentas as previously described (12). The terminal villus-rich fractions were resus-pended in homogenization buffer (20 mM Tris-HCl pH 7.4, 5 mM MgCl_2_, 0.25 M sucrose, 1 mM PMSF, 1 *μ*M aprotinin and 1 *μ*M pepstatin A) and disrupted using a Bioruptor (Cell Disruption Bomb; Parr Instrument, Moline, IL, USA). After 15 min of shaking under high pressure at 1,300 psi on ice, cells in terminal villi were disrupted by passage through a narrow valve. The collected homogenate was centrifuged twice at 4°C, first at 600 x g for 10 min and then at 10,000 x g for 20 min. The supernatant was transferred to ultracentrifuge tubes and centrifuged at 105,000 x g for 1 h at 4°C. The pellets, representing the microsomal fraction, were resuspended in phosphate-buffered saline (PBS) containing 5 mM MgCl_2_ and 1% BSA. Magnetic Dynabeads M280 sheep anti-rabbit IgG (catalog no. 112.04; Invitrogen) was used for immunoprecipitation. The microsomal fraction was pre-incubated with Dynabeads bound to the non-immune rabbit IgG whole molecule (catalog no. 011-000-003; Jackson ImmunoResearch Laboratories) at 4°C overnight to reduce non-specific binding. To capture FCGR2B2-containing vesicles, the pre-incubated microsomal fraction was incubated with Dynabeads and either anti-FCGR2B antibody or the non-immune rabbit IgG as a control at 4°C for 2 h. The beads were then washed 5 times with PBS at room temperature and subjected to the following procedures.

### Transmission electron microscopy of FCGR2B2 compartments

The FCGR2B2 compartments were fixed overnight at room temperature with 2.5% glutaraldehyde and 2% paraformal-dehyde in 0.1 M sodium cacodylate buffer, pH 7.4 containing 0.05% CaCl_2_. The pellet was post-fixed with 2% osmium tetroxide and 1.6% potassium ferrocyanide in 0.1 M sodium cacodylate at room temperature for 30 min. The pellet was dehydrated in ethanol and then embedded in epoxy resin. The sections were electron stained with uranyl acetate and lead citrate and examined under a transmission electron microscope (H-7600; Hitachi) at 80 kV.

### Two-dimensional differential in-gel electrophoresis (2D-DIGE) and mass spectrometry

2D-DIGE was performed as previously described (21). The isolated FCGR2B2 compartments were lysed with thiourea lysis buffer. As a control, an equal volume of the control beads bound to the non-immune rabbit IgG whole molecule was used. The proteins extracted from the purified FCGR2B2 compartments and the control beads were labeled with CyDye DIGE Fluor Cy3 and Cy5 minimal Dyes (GE Healthcare), respectively. The CyDye-labeled proteins and non-labeled microsomal proteins (as a spot reference) were then mixed and subjected to 2D-DIGE. The CyDye-labeled protein spots in the gel were visualized using a Typhoon 9400 imager and analyzed using differential in-gel analysis with DeCyder 2D Differential Analysis Software (both from GE Healthcare). Protein spots that exhibited 2-fold higher intensity in the FCGR2B2 compartments compared to the control were selected as candidate FCGR2B2 compartment-associated proteins. All proteins, including the non-labeled microsomal proteins, on the same gel were then stained with SYPRO Ruby (Takara Bio) to determine the positions of the FCGR2B2 compartment-associated proteins. The eluted proteins were analyzed by liquid chromatography-tandem mass spectrometry (LC-MS/MS) (LCQ DECA, XP Plus; Thermo Finnigan, San Jose, CA, USA). Data were analyzed using Mascot Search on the Matrix Science website (http://www.matrixscience.com/). The specificity of trypsin digestion was used as the cutting enzyme, allowing 3 missed cleavages. Peptide mass tolerance and fragment mass tolerance were set to ±1.2 and ±0.6 Da, respectively. Carbamidomethyl of cysteine was selected as the fixed modification and oxidation of methionine was searched as the variable modification. Identity or extensive homology is indicated for peptides with individual ion scores >42 (p<0.05).

### Statistical analysis

We conducted all the analyses using the SPSS statistical software package (Windows version 20; IBM-SPSS). The significance of between-group differences was assessed using the Student's t-test or ANOVA with Dunnett’s post test, and p-values <0.05 were considered to indicate statistically significant differences.

## Results

### Expression and localization of FCGR2B2 in pFCGR2B2- EGFP-transfected HUVECs

**Figure 2:**
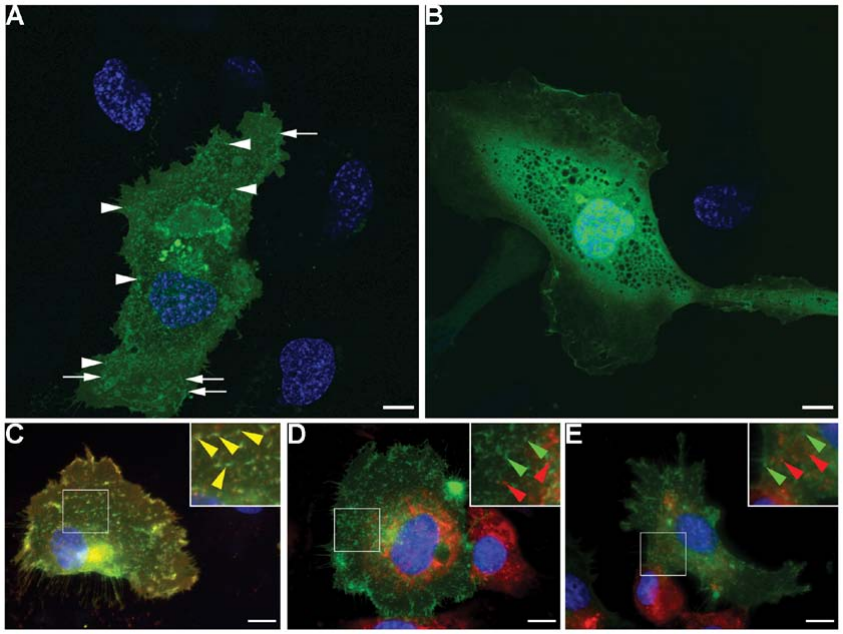
Localization of FCGR2B2 in pFCGR2B2-EGFP-transfected human umbilical vein endothelial cells (HUVECs). (A and B) Representative confocal fluorescence images of HUVECs transfected with either (A) pFCGR2B2-EGFP or (B) pEGFP-N1. (A) In a pFCGR2B2-EGFP-transfected cell, numerous FCGR2B2-EGFP signals (green) are distributed throughout the cytoplasm; green signals are detected as intracellular vesicular (arrowheads) and tubular structures (arrows). The signals are also evident on the Golgi apparatus. (B) By contrast, in a mock-transfected cell, the EGFP signals are diffusely distributed within the cytoplasm and the nuclei. (C–E) Representative fluorescence microscopy images of pFCGR2B2-EGFP-transfected HUVECs immunostained with (C) anti-FCGR2B antibody, (D) anti-von Willebrand factor antibody, or (E) anti-caveolin-1antibody. Alexa Fluor 594-labeled secondary antibodies were used to detect the primary antibodies. Each inset shows a higher magnification view of the region in the white frame. (C) FCGR2B2-EGFP signals (green) and Alexa Fluor 594 signals detected with anti-FCGR2B2 antibody (red) are co-localized (yellow arrowheads). By contrast, FCGR2B2-EGFP signals (green arrowheads) are not co-localized with either (D) von Willebrand factor or (E) caveolin-1 (red arrowheads). Nuclei were counterstained with 4′,6-diamidino-2-phenylindole (DAPI) (blue). Scale bars represent 10 *μ*m. Data are representative of 3 or more independent experiments. FCGR2B2, low-affinity immunoglobulin gamma Fc region receptor IIb2.

In the pFCGR2B2-EGFP-trans-fected HUVECs, fluorescence signals indicating the expression of FCGR2B2 (FCGR2B2-EGFP signals) were transiently detected within 18-48 h after electroporation. In the transfected cells, FCGR2B2-EGFP signals were detected as intracellular vesicular and tubular structures and were widely distributed in the cytoplasm (Fig. 2A). Some signals were also present on the cell surface. The pFCGR2B2-EGFP-transfected HUVECs showed a similar expression of FCGR2B2 in placental endothelial cells *in vivo* as previously reported (11). By contrast, in the mock-transfected pEGFP-N1 cells, the fluorescence signals showed a diffuse distribution throughout the cytoplasm and the nucleus and did not exhibit intracellular structure-specific localization (Fig. 2B).

Subsequently, we wished to investigate whether the localization of the FCGR2B2-EGFP reflected that of the endogenous protein. In the pFCGR2B2-EGFP-transfected HUVECs, the majority of the EGFP signals were co-localized with the Alexa Fluor 594 fluorescence signals detected with an anti-FCGR2B antibody (Fig. 2C), thus suggesting that the EGFP signals can be used to reliably monitor FCGR2B2 in the transfected cells. Although an endogenous endothelial marker, von Willebrand factor, and a caveola marker, caveolin-1, were present in the FCGR2B2-EGFP-expressing HUVECs, they were not co-localized with the FCGR2B2-EGFP signals (Fig. 2D and E). These results are consistent with those of previous *in vivo* findings that the intracellular FCGR2B2 compartments do not overlap with caveolae and secretory granules in the placental endothelium (22). In addition, in order to provide an experimental foundation for bio-imaging analysis, we compared the subcellular localization and IgG binding property of FCGR2B2-EGFP with those of FCGR2B2. Immunocytochemistry revealed that the pFCGR2B2-EGFP-transfected HUVECs showed a localization of FCGR2B2 similar to the pFCGR2B2-transfected cells (Fig. 1D). We also examined the affinities of human IgG for FCGR2B2-EGFP and FCGR2B2 by ELISA (Fig. 1E). FCGR2B2-EGFP showed an almost identical affinity of human IgG for FCGR2B2. Taken together, these results indicate that the pFCGR2B2-EGFP-transfected HUVECs are a useful model for placental endothelial cells *in vitro*.

### Human IgG incorporation from the basolateral surface of FCGR2B2-EGFP-expressing cells

**Figure 3:**
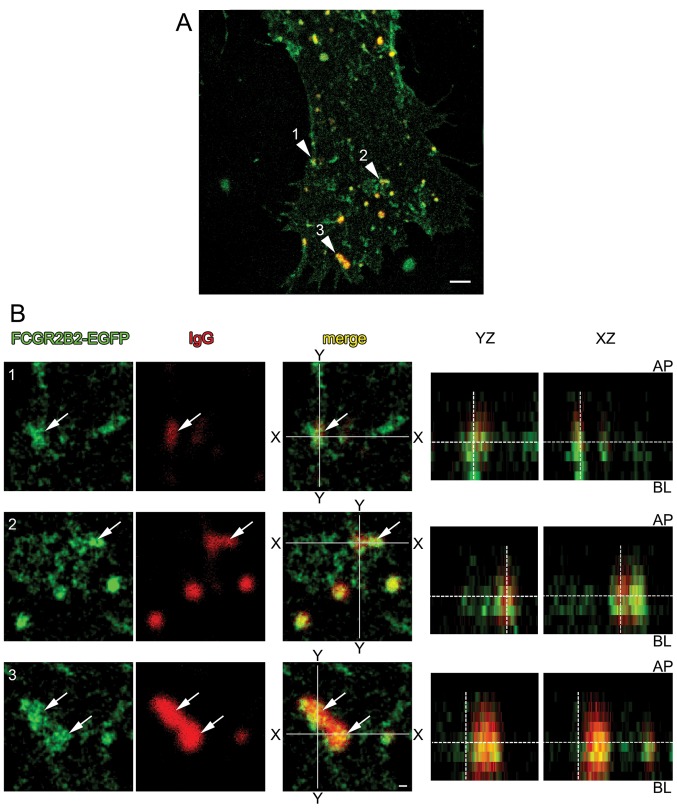
Super-resolution nanoscopic observation of human IgG internalization from the basolateral surface of FCGR2B2-EGFP-expressing human umbilical vein endothelial cells (HUVECs) using stimulated emission-depletion (STED) microscopy. (A) Combined image of FCGR2B2-EGFP (STED in green) and Alexa Fluor 633-IgG (confocal in red) shows that most of the internalized Alexa 633-IgG signals are co-localized with FCGR2B2-EGFP signals. (B) Sets of higher magnification images of 3 regions indicated with numbered arrowheads in (A). Each set is arranged with the following images, from left to right: STED microscopy image showing FCGR2B2-EGFP (FCGR2B2-EGFP), confocal image with Alexa 633-IgG (IgG), the merged image (merge), a virtual slice cut at a vertical line indicated with YY in merged image (YZ), and a virtual slice cut at a horizontal line indicated with XX in merged image (XZ). Apical (AP) and basolateral (BL) sides are evident. Oval and tubular FCGR2B2-EGFP signals are co-localized with Alexa Fluor 633-IgG signals that are incorporated from basolateral surface (arrows). Scale bars represent (A) 2 *μ*m and (B) 200 nm. Three independent experiments were performed and yielded similar results. FCGR2B2, low-affinity immunoglobulin gamma Fc region receptor IIb2.

As maternal IgG is internalized from the basolateral surface of the placental endothelium *in vivo*, we then examined the subcellular IgG incorporation from the basolateral surface of FCGR2B2-EGFP-expressing HUVECs by both STED and confocal imaging. STED microscopy is a technique that uses the non-linear de-excitation of fluorescent dyes to overcome the resolution limit of standard confocal microscopes that is imposed by diffraction (23). Confluent pFCGR2B2-EGFP-transfected HUVECs that were cultured on Transwell filters were exposed to Alexa Fluor 633-labeled IgG (Alexa 633-IgG) from the basolateral surface. Super-resolution imaging with STED and confocal imaging revealed that oval and tubular FCGR2B2-EGFP signals were often found and frequently co-localized with Alexa Fluor 633-IgG signals that were incorporated from the basolateral surface (Fig. 3). By three-dimensional analysis, it appeared that some FCGR2B2-EFGP compartments extended or fused along the axis of apical-basal cell polarity (Fig. 3B, YZ and XZ).

### Internalization and transcytosis of human IgG in the FCGR2B2-EGFP-expressing cells

**Figure 4:**
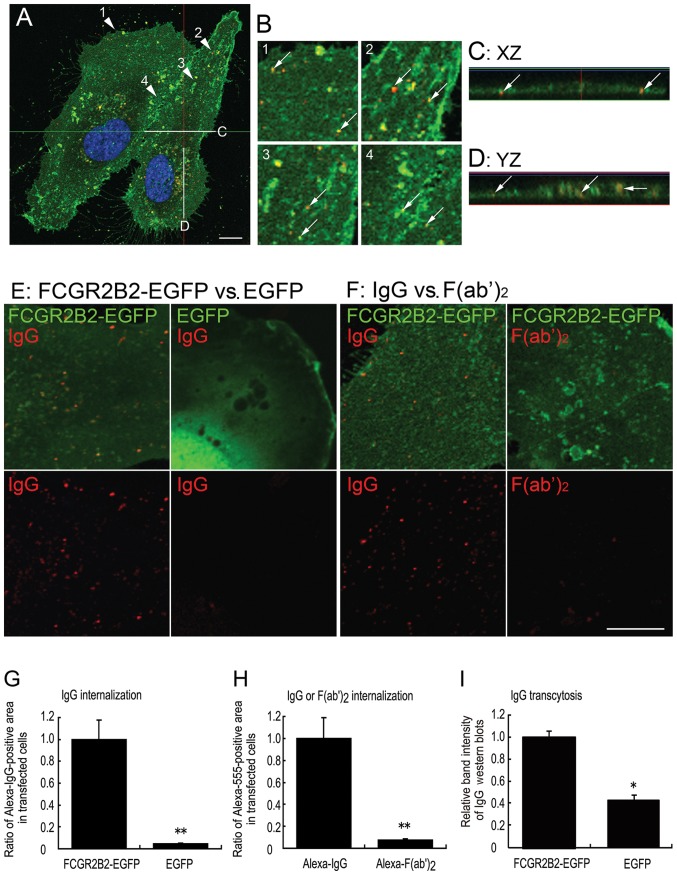
Internalization and transcytosis of human IgG in FCGR2B2-EGFP-expressing cells. (A-D) Representative confocal fluorescence images of internalized Alexa Fluor 555-IgG (red) in FCGR2B2-EGFP (green)-expressing human umbilical vein endothelial cells (HUVECs). (A) A confocal optical slice of 0.6 *μ*m thickness at the position closest to the basal surface. Nuclei were counterstained with 4′,6-diamidino-2-phenylindole (DAPI) (blue). (B) Higher magnification images of the regions indicated with the numbered arrowheads in (A). (C and D) Virtual slices cut at the horizontal gray line denoted by (C and D) in (A). In each virtual slice, the apical surface of the HUVECs is indicated as the upper side. Internalized Alexa Fluor 555-IgG signals appear as small dots in the cells. Co-localization of internalized Alexa Fluor 555-IgG and FCGR2B2-EGFP signals is frequently observed (arrows in B-D). (E) Higher magnification confocal images of Alexa Fluor 555-IgG (red) in FCGR2B2-EGFP- and EGFP (green)-expressing cells. Internalized Alexa Fluor 555-IgG signals appear as small vesicular structures colocalized with FCGR2B2-compartments in a pFCGR2B2-EGFP-transfected cell. By contrast, in an EGFP (mock)-transfected cell, the level of internalized Alexa Fluor 555-IgG is very low. (F) Higher magnification confocal images of Alexa Fluor 555-IgG or Alexa Fluor 555-F(ab′)_2_ fragments (red) in FCGR2B2-EGFP-expressing cells. The internalization of the Alexa Fluor 555-F(ab′)_2_ signal is obviously lower than that of Alexa Fluor 555-IgG. Scale bars are 10 *μ*m. (G and H) Quantification of internalized Alexa Fluor 555-IgG or Alexa Fluor 555-F(ab′)_2_ signals. The area of intracellular Alexa Fluor 555-IgG or Alexa Fluor 555-F(ab′)_2_ signals was quantified. Values are the means ± SE [FCGR2B2-EGFP-expressing cells, n=47; EGFP (mock)-expressing cells, n=48 (G)] and the mean ± SE [Alexa 555-IgG-internalized cells, n=43; Alexa 555-F(ab′)_2_-internalized cells, n=48) (H)]. ^**^p<0.01, Student’s t-test. Three or more experiments were performed and yielded similar results. (I) Quantification of transcytosed, biotin-labeled human IgG by western blot analysis. The band intensities of the heavy chain of the transcytosed, biotin-labeled IgG are significantly higher in pFCGR2B2-EGFP-transfected cells than in EGFP (mock)-transfected cells (^*^p<0.05, Student's t-test). The data are the means from 3 measurements; error bars represent the means ± SD. FCGR2B2, low-affinity immunoglobulin gamma Fc region receptor IIb2.

The ability of FCGR2B2-EGFP-expressing HUVECs to incorporate human IgG was evaluated by monitoring the fluorescence signals in the cells exposed to Alexa Fluor 555-labeled human IgG (Alexa 555-IgG). In the cells, the majority of the internalized Alexa 555-IgG signals were co-localized with the FCGR2B2-EGFP signals (Fig. 4A–D). Internalized Alexa 555-IgG was more easily detected as small dots of fluorescence in the pFCGR2B2-EGFP-transfected cells than in the EGFP (mock)-transfected cells (Fig. 4E). The ability of the FCGR2B2-EGFP-expressing cells to incorporate IgG was evaluated by comparing the ratios of the Alexa 555-positive area to the total cellular area in the pFCGR2B2-EGFP-transfected cells (n=47) and mock-transfected cells (n=48); this ratio was significantly higher in the pFCGR2B2-EGFP-transfected cells than in the mock-transfected cells (P<0.01) (Fig. 4G). Furthermore, to validate the Fc fragment-specific internalization, FCGR2B2-EGFP-expressing cells were incubated with equimolar concentrations of IgG or F(ab′)_2_ fragments (Fig. 4F). The ratios of the Alexa 555-IgG and Alexa 555-labeled-F(ab′)_2_ fragment [Alexa 555-F(ab′)_2_] positive areas were measured in 43 and 48 cells, respectively. While the Alexa 555-F(ab′)_2_ was detected in the FCGR2B2-EGFP-expressing cells (Fig. 4F), the ratio of Alexa 555-F(ab′)_2_ was significantly lower than the ratio of Alexa 555-IgG (P<0.01) (Fig. 4H).

Subsequently, we aimed to investigate the ability of the pFCGR2B2-EGFP-transfected HUVECs to transcytose IgG. Biotin-labeled human IgG was added to in the basolateral donor chamber. After 24 h, transcytosed, biotin-labeled human IgG in the apical receiver chamber was detected by western blot analysis. The band intensities of the heavy chain of the transcytosed, biotin-labeled IgG were significantly higher in the pFCGR2B2-EGFP-transfected cells than in the mock-transfected cells (P<0.05) (Fig. 4I). Taken together, these results suggest that the IgG internalization and transcytosis observed in this study was caused by the enforced expression of FCGR2B2 in the HUVECs and that it was an Fc fragment-specific event.

### IgG internalization and transcytosis in FCGR2B2-EGFP-expressing cells that are FCGRT-deficient

**Figure 5:**
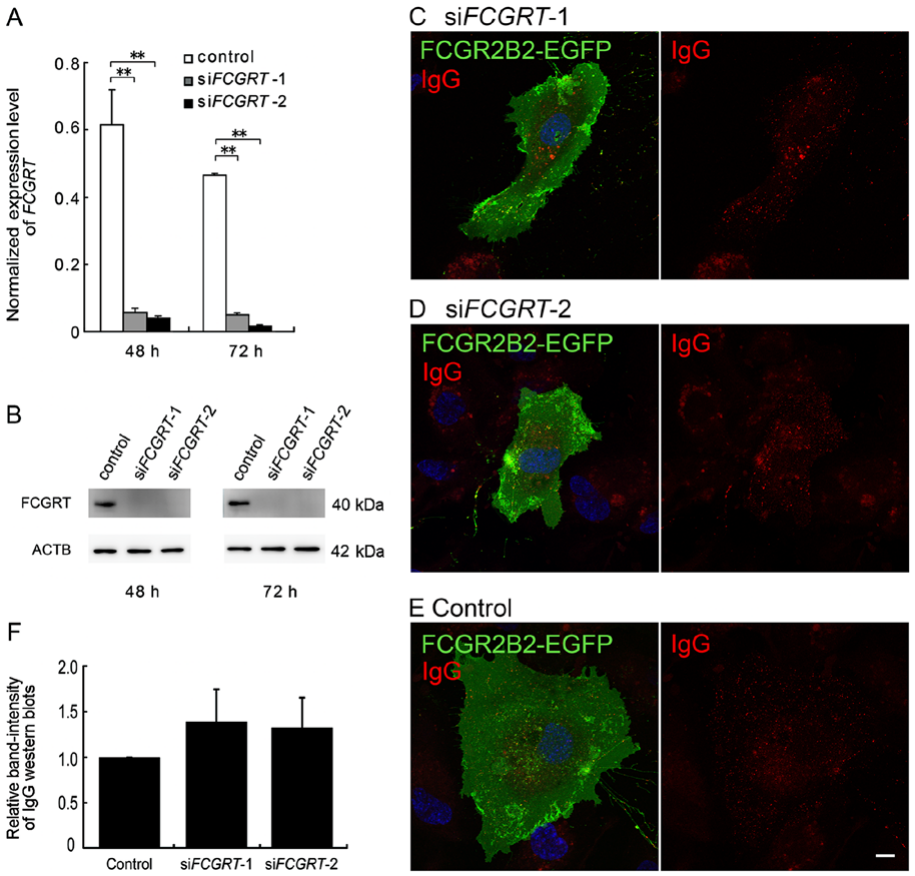
IgG internalization and transcytosis in FCGR2B2-EGFP-expressing cells following the downregulation of FCGRT. (A and B) The efficiency of siRNA-mediated knockdown of *FCGRT* in human umbilical vein endothelial cells (HUVECs). (A) RT-qPCR reveals that both si*FCGRT-1* and si*FCGRT-2* significantly reduce the *FCGRT* mRNA level in the HUVECs after 48 and 72 h. The data are the means from 3 measurements. Error bars represent the means ± SD. ^**^p<0.01, one-way ANOVA with Dunnett’s post test. (B) Western blot analysis reveals that the FCGRT protein levels in the HUVECs are markedly downregulated at both 48 and 72 h after transfection of siRNAs. (C-E) Representative confocal fluorescence images of internalized Alexa Fluor 555-IgG in FCGR2B2-EGFP-expressing HUVECs after FCGRT knockdown. After 18–24 h to transfect with p*FCGR2B2-EGFP*, HUVECs were transfected with siRNAs and incubated for another 18 h. Transfection of (C) si*FCGRT-1* and (D) si*FCGRT-2* do not affect the expression and distribution of FCGR2B2-EGFP or the internalization of Alexa 555-IgG signals compared to the control siRNA (E). Nuclei were counterstained with 4′,6-diamidino-2-phenylindole (DAPI) (blue). Scale bar represents 10 *μ*m. Three independent experiments were performed with similar results. (F) Quantification of transcytosed, biotin-labeled human IgG by western blot analysis. No significant differences in the band intensities of the heavy chain of the transcytosed, biotin-labeled IgG are identified between *FCGRT*-knockdown (si*FCGRT-1* and si*FCGRT-2*) and mock (control) cells (one-way ANOVA). The data are the mean from 3 measurements; error bars represent the means ± SD. FCGR2B2, low-affinity immunoglobulin gamma Fc region receptor IIb2.

HUVECs also express FCGRT; thus, we examined the effects of endogenous FCGRT on IgG incorporation by siRNA-mediated knockdown. We constructed 2 types of siRNA duplexes against *FCGRT* (designated as si*FCGRT*-1 and si*FCGRT*-2). First, we evaluated the efficiency of the siRNA-mediated knockdown using RT-qPCR and western blot analysis. After 48 h of siRNA transfection, the mRNA expression level of *FCGRT* was 9.2 and 6.6% of the control cell levels with si*FCGRT*-1 and si*FCGRT*-2, respectively. After 72 h, the expression level was 10.6% (si*FCGRT*-1) and 3.4% (si*FCGRT*-2) of the control (Fig. 5A). Furthermore, the protein expression of FCGRT was markedly decreased by si*FCGRT*-1 and si*FCGRT*-2 at both 48 and 72 h, as determined by western blot analysis (Fig. 5B). As these siRNAs efficiently suppressed the expression of both the *FCGRT* mRNA and FCGRT protein, they can be used for subsequent experiments to investigate the effect of endogenous FCGRT on IgG incorporation in FCGR2B2-EGFP-expressing HUVECs.

After 18-24 h of transfection with p*FCGR2B2-EGFP*, the HUVECs were transfected with siRNAs and incubated for a further 18 h. The transfection of *siFCGRT*-1 and *siFCGRT*-2 did not affect the expression and distribution of FCGR2B2-EGFP (Fig. 5C–E). The internalization of Alexa 555-IgG was not altered in the cells in which *FCGRT* had been knocked down (Fig. 5C and D). Furthermore, we investigated the ability of the cells in which *FCGRT* had been knocked down to tran-scytose IgG using the above-mentioned biotin-labeled human IgG. No significant differences in the band intensities of the heavy chain of the transcytosed, biotin-labeled IgG were identified between the cells in which *FCGRT* had been knocked down and the mock cells (Fig. 5F). Thus, our results suggested that the IgG internalization and transcytosis were mediated primarily by the enforced expression of FCGR2B2 rather than that of endogenous FCGRT in the HUVECs.

### Identification of FCGR2B2 compartment-associated proteins from the human placenta

**Figure 6:**
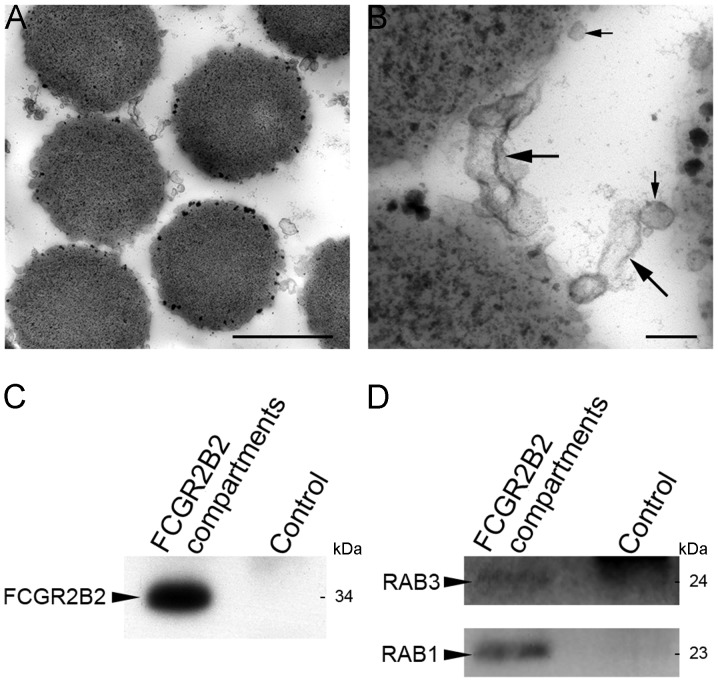
Validation of isolated FCGR2B2 compartments. FCGR2B2 compartments were isolated from human full-term placenta. (A and B) Electron microscopy analysis reveals that on the surface of the magnetic beads bound to the anti-FCGR2B antibody, round (small arrows) and tubular (large arrows) membranous compartments are visible. These morphological characteristics are similar to FCGR2B2 compartments previously observed in human placental endothelial cells *in vivo* (11). Scale bars represent (A) 2 *μ*m and (B) 200 nm. (C and D) Western blot analysis confirms that FCGR2B2 compartments are specifically captured by the beads coated with the anti-FCGR2B antibody (C). RAB3 and RAB1 proteins are detected on the FCGR2B2 compartments (D). The broad smear of the control fraction in the RAB3 panel is considered to be a non-specific signal because its molecular weight is >24 kDa. FCGR2B2, low-affinity immunoglobulin gamma Fc region receptor IIb2.

We performed a proteomic analysis to identify FCGR2B2 compartment-associated proteins, some of which may be involved in the regulation of the trafficking of FCGR2B2 compartments. FCGR2B2 compartments were isolated from human placental terminal villus-rich fractions by immunoprecipitation using magnetic beads bound to a FCGR2B-specific antibody as described in the Materials and methods. Imaging by electron microscopy revealed that FCGR2B2 compartments were round, oval and tubular membranous components (Fig. 6A and B). These morphological characteristics were similar to those observed by the immunoelectron microscopy of FCGR2B2 compartments in placental endothelial cells (11). Western blot analysis revealed that the FCGR2B2 compartments were specifically captured on the beads coated with the anti-FCGR2B antibody (Fig. 6C).

Proteomic analysis of the FCGR2B fraction was performed by 2D-DIGE. Twenty protein spots showed a >2-fold higher volume ratio in the FCGR2B2 compartment fraction compared with the control fraction. However, 4 of these spots were part of a horizontal streaking tail; thus, they were not subjected to further analyses. Of the 16 spots, 2 minor spots could not be separated or detected on gels for protein identification. The remaining 14 spots were isolated and analyzed by LC-MS/MS. The FCGR2B2 compartment-associated proteins identified by LC-MS/MS are summarized in Table I. Of note, LC-MS/MS revealed that these proteins contained a number of RAB family proteins.

**Figure 7:**
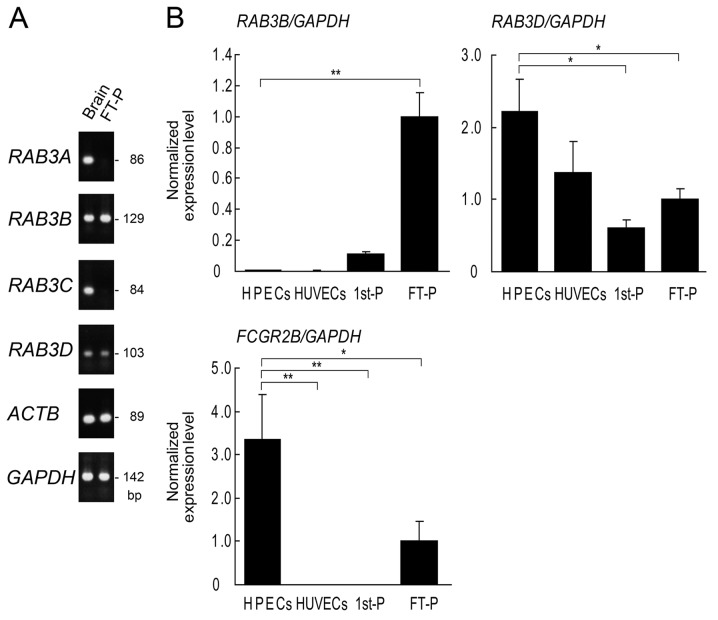
RT-qPCR of *RAB3* isoforms in human placental tissues and isolated endothelial cells. (A) RT-qPCR of full-term placentas (FT-P) and human brain (Brain) as a positive control. *RAB3A* and *RAB3C* are absent in FT-P. Other isoforms of *RAB3*, *RAB3B* and *RAB3D*, are expressed in FT-P. (B) RT-qPCR of human placental endothelial cells (HPECs), human umbilical vein endothelial cells (HUVECs), first-trimester placentas (1st-P) and FT-P. HPECs and HUVECs were isolated simultaneously from FT-P and umbilical cords, respectively. *FCGR2B* is highly expressed in HPECs, but is not expressed in HUVECs. *RAB3D* is highly expressed in HPECs compared to 1st-P, FT-P, and HUVEC. Expression levels were normalized to *GAPDH*. Relative expression levels in FT-P were assigned a value of 1. Data are indicated as the mean from the results using 3 pregnant women. Error bars represent the means ± SD. ^*^p<0.05 and ^**^p<0.01, one-way ANOVA with Dunnett’s post test. FCGR2B2, low-affinity immunoglobulin gamma Fc region receptor IIb2.

**Figure 8:**
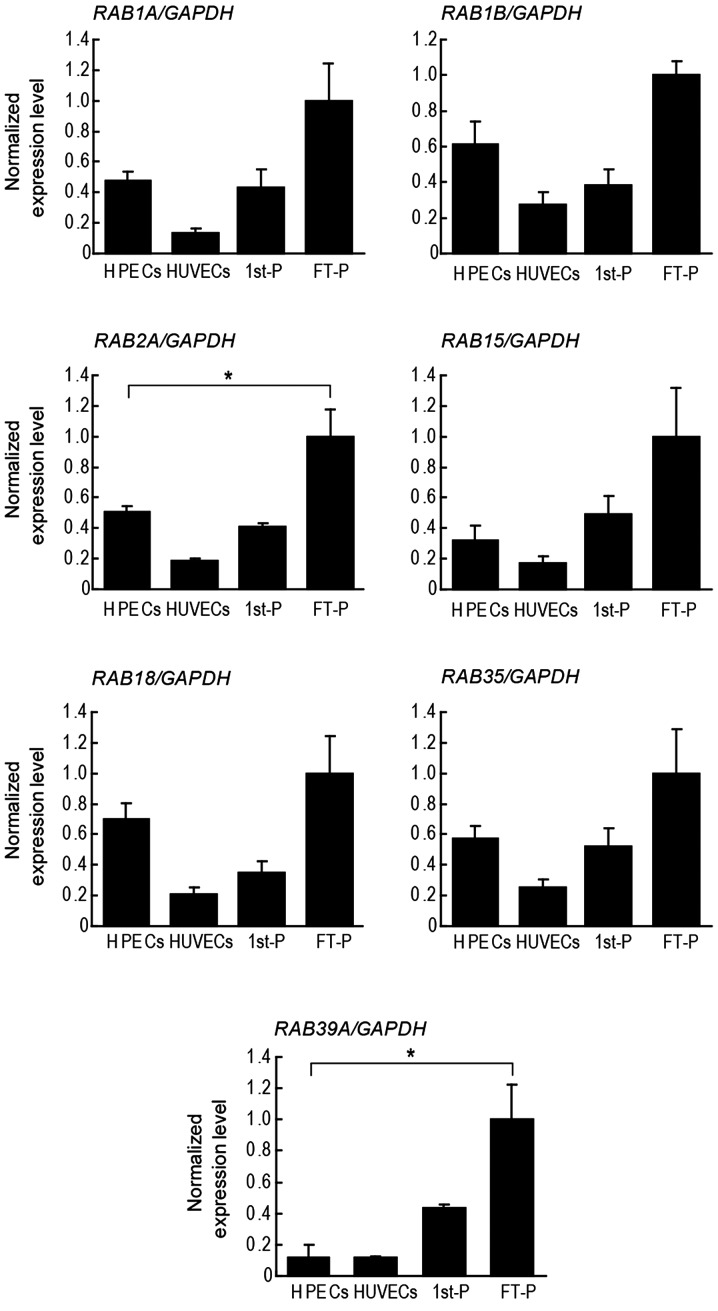
RT-qPCR of mRNAs encoding other RAB family proteins in human placental tissues and isolated endothelial cells. RT-qPCR of RAB mRNAs in human placentas and isolated endothelial cells. The expression levels of RAB mRNAs (*RAB1A*, *RAB1B*, *RAB2A*, *RAB12*, *RAB18*, *RAB35* and *RAB39*) identified from isolated FCGR2B2 compartments by proteomic analysis were examined in human placental endothelial cells (HPECs), human umbilical vein endothelial cells (HUVECs), first-trimester placentas (1st-P) and full-term placentas (FT-P). The expression levels were normalized to GAPDH. The relative expression levels in FT-P were assigned a value of 1. Data are the mean from the results using 3 pregnant women. Error bars represent the means ± SD. ^*^p<0.05, one-way ANOVA with Dunnett's post test. FCGR2B2, low-affinity immunoglobulin gamma Fc region receptor IIb2.

Subsequently, we examined the expression of some of the RAB family proteins identified by LC-MS/MS in the isolated FCGR2B2 compartments (Table I). Western blot analysis revealed that both RAB3 and RAB1 were detectable in the FCGR2B2 compartments (Fig. 6D). However, specific antibodies against the other RABs identified by LC-MS/MS were not commercially available for use in western blot analysis. In order to examine which isoforms may be associated with the FCGR2B2 compartments and which isoforms may be highly expressed in placental endothelial cells, we investigated the mRNA expression levels of each isoform (i.e., *RAB1A*, *RAB1B*, *RAB2A*, *RAB3A*, *RAB3B*, *RAB3D*, *RAB15*, *RAB18*, *RAB35* and *RAB39A*) in placental tissues (first-trimester and full-term placentas) and isolated endothelial cells (placental endothelial cells and HUVECs). At first, we examined 3 *RAB3* isoforms, *RAB3A*, *RAB3B* and *RAB3D*, and another highly homologous *RAB3* isoform, RAB3C. RT-qPCR revealed that *RAB3A* and *RAB3C* were absent in the full-term placentas (Fig. 7A). The mRNA expression levels of *RAB3B*, *RAB3D*, and other RABs were subsequently compared among first-trimester placentas, full-term placentas, placental endothelial cells and HUVECs by RT-qPCR. *RAB1A*, *RAB1B*, *RAB2A*, *RAB3D*, *RAB15*, *RAB18*, *RAB35* and *RAB39A* were expressed in all the samples examined; *RAB3B* was detected in the placental tissues, but not in the endothelial cells (Figs. 7B and 8). Among these RAB mRNAs, only *RAB3D* was expressed predominantly in the placental endothelial cells (Fig. 7B). Therefore, we focused on RAB3D as a candidate FCGR2B2 compartment-associated protein to investigate its involvement in the formation and/or intracellular dynamics of FCGR2B2 compartments.

### Association of the downregulation of RAB3D with FCGR2B2 compartments using gene specific siRNAs

**Figure 9:**
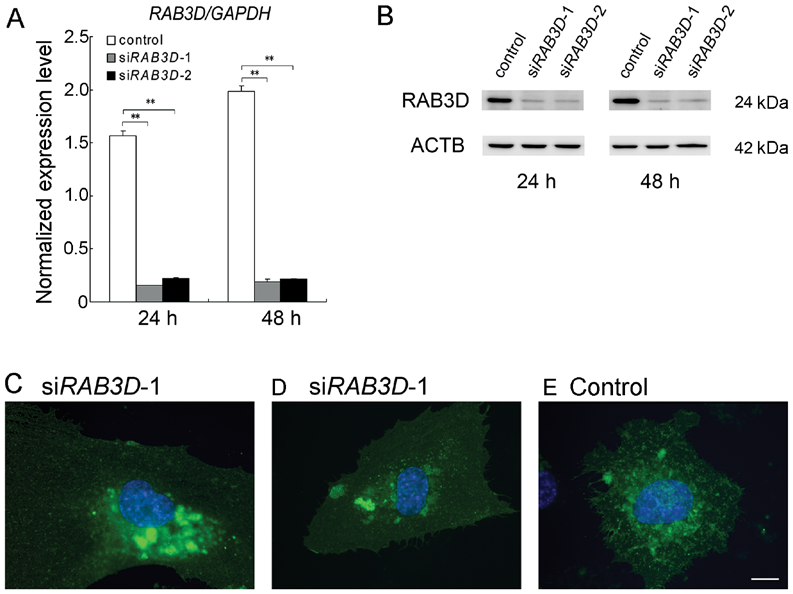
Intracellular dynamics of FCGR2B2 compartments in cells in which RAB3D was knocked down. (A and B) Validation of the siRNA-mediated knockdown efficiency of *RAB3D* in human umbilical vein endothelial cells (HUVECs). (A) RT-qPCR reveals that both si*RAB3D-1* and si*RAB3D-2* efficiently reduce the *RAB3D* mRNA level in the HUVECs after 24 and 48 h. Data are the mean from 3 measurements. Error bars represent the means ± SD. ^**^p<0.01, one-way ANOVA with Dunnett’s post test. (B) Western blot analysis reveals that RAB3D protein levels in the HUVECs are downregulated at both 24 and 48 h after transfection of siRNAs. (C-E) Representative fluorescence images of FCGR2B2-EGFP (green) with 4′,6-diamidino-2-phenylindole (DAPI) (blue) in HUVECs transfected with 2 distinct siRNAs directed against *RAB3D* mRNA [(C) si*RAB3D-1* and (D) si*RAB3D-2*] and (E) control non-targeting siRNA. After 18–24 h of transfection with pFCGR2B2-EGFP, the HUVECs were transfected with siRNAs and incubated for a further 18 h. (C and D) RAB3D knockdown results in the marked reduction of FCGR2B2-EGFP signals at the cell periphery and accumulation of FCGR2B2-EGFP as large vesicular compartments in the juxtanuclear area. Scale bar represents 10 *μ*m. FCGR2B2, low-affinity immunoglobulin gamma Fc region receptor IIb2.

We observed FCGR2B2-EGFP expression in the cells in which *RAB3D* expression was downregulated by siRNA-mediated knock down. We constructed 2 types of siRNA duplexes against *RAB3D* (designated as si*RAB3D*-1 and si*RAB3D*-2). First, we evaluated the efficiency of the siRNA-mediated knockdown by RT-qPCR. In the HUVECs, si*RAB3D*-1 and si*RAB3D*-2 inhibited the expression of each mRNA by 9.8 and 14.2% after 24 h and by 9.5 and 10.7% after 48 h, respectively (Fig. 9A). The protein level of RAB3D was also reduced by siRNA-mediated knockdown (Fig. 9B).

We cultured the pFCGR2B2-EGFP-transfected HUVECs until an EGFP signal could be observed and then treated them with the si*RAB3Ds*. Following *RAB3D* knockdown, we observed a redistribution of the FCGR2B2-EGFP signals in the transfected cells; the FCGR2B2-EGFP signals were markedly reduced at the cell periphery and accumulated as large vesicular compartments in the juxtanuclear area (Fig. 9C and D). These results suggest that RAB3D participates in the formation of FCGR2B2 compartments in the transfected cells.

## Discussion

In this study, we performed bio-imaging analysis of IgG trafficking in FCGR2B2-transfected HUVECs and demonstrated that FCGR2B2 compartments were involved in IgG transport following basolateral IgG internalization in the transfected cells. Maternal IgG is transferred across two cell layers of the human placenta, the syncytiotrophoblast and the fetal placental endothelium. In the syncytiotrophoblast, the maternal IgG incorporated by non-specific pinocytosis is protected by FCGRT in endosomes and is subsequently transcytosed to the basolateral surface of the cell, i.e., the placental villous stroma. Therefore, the villous stroma contains large amounts of maternal IgG. It is important to understand the mechanisms through which the maternal IgG accumulated in the villous stroma is transported across the fetal endothelium, the second placental barrier. FCGR2B2 is abundantly and exclusively expressed in the placental endothelium (10,11). We have previously suggested that a distinct FCGR2B2-containing organelle in the placental endothelium mediates the transcytosis of maternal IgG (11). However, it is unclear as to how IgG negotiates the placental endothelial cells. We can envision at least three possible mechanisms: passive movement along a constitutive endocytic pathway, movement down an IgG concentration gradient, or transfer of IgG mediated by FCGR2B2. Thus, we developed an *in vitro* model system for the analysis of IgG transport in the placental endothelium. We transfected pFCGR2B2-EGFP vectors into HUVECs and generated FCGR2B2-EGFP-expressing fetal endothelial cells as mimics of placental endothelial cells. Indeed, EGFP signals indicating FCGR2B2 were distributed in vesicular and tubular compartments that were distinct from caveolae and secretory granules. The majority of the internalized IgG was associated with FCGR2B2-EGFP-positive compartments. Our *in vitro* findings are consistent with our previous *in vivo* results showing that the FCGR2B2 compartments are intracellular organelles of the placental endothelium, not associated with caveolae or secretory granules (11).

FCGR2B is thought to bind preferentially to IgG in the form of immune complexes (24,25). However, crystallographic studies of FCGR2A and FCGR2B have suggested that the receptor dimers can also bind to monomeric IgG (26,27). Moreover, functional mapping using synthetic peptides corresponding to stretches of the IgG Fc peptides has also indicated that the IgG1 CH2 domain binds to both soluble and cell surface-expressed forms of FCGR2B (14). FCGR2B2 mediates endocytosis and transcytosis, while its companion isoform, FCGR2B1, fails to enter endocytic compartments (28,29). Of note, FCGR2B is typically undetectable in endothelial cells in adult human tissues apart from the placental endothelium and the hepatic sinusoid, and both organs express FCGR2B2 and not FCGR2B1. Mousavi *et al* demonstrated that FCGR2B2 expression in rat hepatic sinusoidal endothelial cells serves as a recycling receptor (30); however, their study focused on the receptor-mediating endocytosis of immune complexes. Taken together with previous observations, the present study lends support to the idea that FCGR2B2 plays an important role in the intracellular trafficking of IgG in the human placental endothelium.

Based on our data, it can also be concluded that the internalization and transcytosis of IgG is accelerated by FCGR2B2 rather than FCGRT in our *in vitro* model of the human placental endothelium. Although FCGRT transports both IgG and albumin (31), albumin cannot easily be transported like maternal IgG across the human placenta (32,33). One possible explanation of the selective transfer of IgG across the placental barrier for our findings may be that FCGRT of the syncytiotrophoblast transports both ligands but FCGR2B2 of the placental endothelium allows only IgG to pass. However, we have not illustrated the whole picture of Fc receptors and their associated regulatory factors involved in IgG transcytosis in the human placental endothelium. FCGRT is expressed not only in the syncytiotrophoblast, but also in adult endothelial cells; it regulates the serum levels of IgG (34,35). There is still limited knowledge concerning intracellular trafficking in endothelial cells. It would be of interest to investigate whether FCGRT cooperates with FCGR2B2 to transcytose IgG in the human placental endothelium by imaging analysis. However, the anti-FCGRT antibody generated in this study could not work well in immunocytochemistry. The involvement of FCGRT in IgG transcytosis in the placental endothelium remains an issue.

The mouse placenta does not transport IgG; rather, the yolk sac is the organ responsible for maternal-fetal IgG transport in the mouse. Maternal IgG is transported across the mouse yolk sac endoderm by FCGRT (36). The yolk sac also expresses FCGR2B2. However, FCGR2B2 is not present in the yolk sac in endothelial cells; non-endothelial FCGR2B2 is not required for the transport of IgG to the fetus (37,38). The vascular architecture of the human placenta is quite distinct from that of the mouse yolk sac, and FCGR2B2 is expressed in different cell types along the IgG transport pathway between the two species. In addition, the carboxy-terminal sequence involved in phagocytosis by murine FCGR2B2 is not conserved in human FCGR2B2 (39). This may also be a difference in the behavior of human and mouse FCGR2B2 which could impinge on the intracellular trafficking. Therefore, it is unlikely that the yolk sac is suitable for study as a model for human IgG transport across placental endothelial cells. *In vivo* studies using Fc receptor gene-knockout nonhuman primate models are important future directions.

To identify molecules that regulate FCGR2B2 compartment trafficking in the human placenta, proteomic analysis of FCGR2B2 compartments isolated from human placental tissues was performed. A large number of FCGR2B2 compartment-associated proteins was identified, including RAB family proteins, which have also been identified in several previous proteomic studies on synaptic vesicles (40), insulin secretory granules (41), endocytic vesicles (42), clathrin-coated vesicles (43) and clathrin-independent carriers (44). RAB proteins are small GTPases and members of the Ras superfamily of monomeric G proteins. RAB proteins regulate each step in the membrane trafficking process: vesicle or tubule formation by budding from donor membranes, intracellular vesicle delivery, vesicle tethering and fusion to specific acceptor membranes with several specific RAB effectors (45). The localization and novel functional aspects of RAB proteins have been revealed (46). In this study, we focused on RAB3D as only *RAB3D* was expressed predominantly in placental endothelial cells. Using siRNA analysis, we found that RAB3D was involved in the intracellular dynamics of FCGR2B2 compartment formation. RAB3 proteins, known as secretory RABs, are primarily local-ized in secretory vesicles and intracellular granules together with several other RABs and are involved in the regulation of each step of exocytosis with their effectors (47). RAB3D plays an important role in the maturation and/or maintenance of secretory granules (48-51). RAB3D also participates in the regulation of the apically directed transcytosis in rat hepato-cytes (52). In endothelial cells, RAB3D regulates the secretion of von Willebrand factor that is stored in Weibel-Palade bodies, endothelial-specific secretory granules (53). While FCGR2B2 compartments were distinct from Weibel-Palade bodies in FCGR2B2-EGFP-expressing HUVECs (Fig. 2D), RAB3D may serve as an important regulator for intracellular trafficking of IgG, as well as secretory proteins in placental endothelial cells. Although we focused on RAB3D in this study, other RAB proteins were also expressed in placental endothelial cells. This study did not fully explore the regulation of intracellular trafficking and dynamics of FCGR2B2 compartments by RAB proteins. Further functional studies of the FCGR2B2 compartment-associated RAB proteins are required to elucidate the detailed mechanisms of maternal-fetal IgG transport in the human placental endothelium.

In conclusion, in this study, we investigated the dynamics and properties of FCGR2B2 compartments for IgG trafficking using an *in vitro* model of placental endothelial cells. Our data suggest the involvement of FCGR2B2 in IgG transcytosis of the human placental endothelium, thus providing new insight into the mechanism through which IgG crosses the human placenta.

## Figures and Tables

**Table I:** FCGR2B2 compartment-associated proteins identified by LC-MS/MS.

GI no.	Gene symbol	Protein name	Fold change	Western blot analysis of RAB proteins
4504517	*HSPB1*	Heat shock 27 kDa protein 1	3.03	
20147713	*RALA*	Ras-related protein Ral-A (precursor)	3.03	(−)
4758984	*RAB11 A*	Ras-related protein Rab-11A	3.03	(+/−)^a^
181178	*CTSB*	Lysosomal proteinase cathepsin B	2.48, 2.02	
182518	*FTL*	Ferritin light subunit	2.48	
4504747	*ITGA3*	Integrin alpha 3 isoform a precursor	2.22	
119610452	*MYH10*	Myosin, heavy polypeptide 10, non-muscle, isoform CRA_a	2.22	
32490572	*EPB41L3*	Erythrocyte membrane protein band 4.1-like 3	2.22	
558436	*DLG1*	Homolog of *Drosophila* discs large protein, isoform 2	2.22	
115298659	*SPTA 1*	Spectrin, alpha, erythrocytic 1	2.22	
5729877	*HSPA 8*	Heat shock 70 kDa protein 8 isoform 1	2.22, 2.05	
71773329	*ANXA6*	Annexin VI isoform 1	2.22	
14585873	*DYNC1I2*	Cytoplasmic dynein intermediate chain	2.22	
2352945		Smooth muscle myosin heavy chain SM2	2.22	
|2851393	*POR*	NADPH-cytochrome P450 reductase	2.22	
7706706	*SNX9*	Sorting nexin 9	2.22, 2.05	
4506467	*RDX*	Radixin	2.22	
340217	*VIL2*	Cytovillin 2	2.22	
13569962	*RAB1B*	RAB1B, member RAS oncogene family	2.19, 2.14	(+)^b^
229451		Placental lactogen	2.19, 2.14	
4758988	*RAB1A*	RAB1A, member RAS oncogene family	2.19, 2.14	(+)^b^
5803135	*RAB35*	RAB35, member RAS oncogene family	2.19, 2.14, 2.12	ND
38371739	*RAB15*	RAB15, member RAS onocogene family	2.19, 2.12	ND
13786129	*RAB33B*	RAB33B, member RAS oncogene family	2.19, 2.12	(+/−)
1491714	*RAB39A*	Rab-related GTP-binding protein, Rab39A	2.19, 2.12	ND
1710248	*ERP5*	Protein disulfde isomerase-related protein 5	2.17	
550062	*RAB2*	GTP-binding protein, RAB2A	2.14	ND
10880989	*RAB18*	RAB18, member RAS oncogene family	2.14	ND
27734452	*RAB15*	Ras-related protein Rab-15	2.14	ND
4758988	*RAB1A*	RAB1A, member RAS oncogene family	2.12	(+)^b^
4506365	*RAB2A*	RAB2A, member RAS oncogene family	2.12	ND
4759000	*RAB3D*	RAB3D, member RAS oncogene family	2.12, 2.02	(+)^c^
1060888	*PSMD2*	Human 26S proteasome subunit p97	2.06	
179468	*HSD3B*	3-beta-Hydroxysteroid dehydrogenase	2.05	
4505257	*MSN*	Moesin	2.05	
4758304	*PDIA4*	Protein disulfde isomerase-associated 4	2.05	
6470150	*HSPA 5*	BiP protein, heat shock 70 kDa protein 5	2.03	
19923750	*RAB3B*	RAB3B, member RAS oncogene family	2.02	(+)^c^
4506367	*RAB3A*	RAB3A, member RAS oncogene family	2.02	(+)^c^
20178293	*KRT7*	Cytokeratin-7	2.02	
4759140	*SLC9A3R1*	Solute carrier family 9, isoform 3 regulator 1	2.02	
178027	*ACTA 3*	Alpha-actin	2.02	

a RAB11A was detected using mouse anti-human RAB11 antibody.

b RAB1 isoforms were detected using rabbit anti-human RAB1 antiserum.

c RAB3 isoforms were detected using rabbit anti-human RAB3 antiserum. FCGR2B2, low-affinity immunoglobulin gamma Fc region receptor IIb2; LC-MS/MS, liquid chromatography-tandem mass spectrometry; +, positive; +/−, faint; −, no band; ND, not determined.
